# Nuclear filaments: role in chromosomal positioning and gene expression

**DOI:** 10.1080/19491034.2020.1769445

**Published:** 2020-05-26

**Authors:** Manindra Bera, Kaushik Sengupta

**Affiliations:** a Biophysics and Structural Genomics Division, Saha Institute of Nuclear Physics, Kolkata, India; b Department of Cell Biology, Yale University School of Medicine, Connecticut, New Haven, USA; c Homi Bhabha National Institute, Mumbai, India

**Keywords:** Nuclear lamins, intermediate Filaments, laminopathies, inter/Intra-Chromosomal Contacts, loop-Clusters, chromosomal Positioning, actin, myosin

## Abstract

Nuclear lamins form an elastic meshwork underlying the inner nuclear membrane and provide mechanical rigidity to the nucleus and maintain shape. Lamins also maintain chromosome positioning and play important roles in several nuclear processes like replication, DNA damage repair, transcription, and epigenetic modifications. LMNA mutations affect cardiac tissue, muscle tissues, adipose tissues to precipitate several diseases collectively termed as laminopathies. However, the rationale behind LMNA mutations and laminopathies continues to elude scientists. During interphase, several chromosomes form inter/intrachromosomal contacts inside nucleoplasm and several chromosomal loops also stretch out to make a ‘loop-cluster’ which are key players to regulate gene expressions. In this perspective, we have proposed that the lamin network in tandem with nuclear actin and myosin provide mechanical rigidity to the chromosomal contacts and facilitate loop-clusters movements. LMNA mutations thus might perturb the landscape of chromosomal contacts or loop-clusters positioning which can impair gene expression profile.

## Introduction

Eukaryotic cells developed a filamentous protein network to balance the progressive enlargement coupled with acquiring of organellar systems. Eukaryotes also developed a filamentous system to maintain their size, shape and functions. Eukaryotes evolved three major filamentous systems, namely, microfilaments/actin, microtubule and intermediate filaments (IFs) to maintain shape, size, rigidity and proper functioning of each cell. Active transport along the microtubule and actin filaments involving numerous proteins provides precise solutions to diffusion rate and physiological relevance. Brownian diffusion inside the cell varies significantly depending on the size of the molecules; for example, in ~1 cm long neuron, macromolecules with ~10 μm^2^/s diffusion coefficient can take about 2 weeks to travel across one end to another solely depending on Brownian diffusion. On the contrary, the same macromolecule using active diffusion along the microtubule takes about 2–3 hours to travel the same distance which makes it physiologically relevant [1]. Actin and microtubules are conserved throughout the evolution, whereas intermediate filaments evolved into several different types through gene duplication [2–4]. Intermediate filaments have been majorly classified into five different categories [5]. All intermediate filament proteins with the sole exception of lamins are resident in the cytoplasm [6]. Lamins are nuclear intermediate filament proteins that are targeted inside the nucleus by the nuclear localization signal (NLS) and are indispensable in maintaining nuclear shape, size and homeostasis [7]. Along with lamins, actins also form very small and transient filamentous structures inside the nucleus during interphase [8,9]. Tubulin forms filaments during cell division for faithful chromosome segregation [10]. Although lamins are primarily structural proteins involved in maintaining nuclear shape and rigidity, several reports suggest a strong correlation between their filamentous structure and gene expression [11–13]. In this review, we will focus on lamins/nuclear actin and provide a different perspective in this article for their role in chromosome organization, gene expression and subsequently discuss their implications in laminopathies.

## Nuclear lamin

Nuclear lamins form a filamentous network underlying the inner nuclear membrane and provide mechanical rigidity to the nucleus [14]. The mechanical scaffolding of lamina helps to retain nuclear shape and morphology. In 1966, Fawcett first observed a filamentous network underlying the inner nuclear membrane which he termed as ‘lamina’[15] and a few years later Gerace & Blobel successfully identified the proteins involved in this lamina formation [16,17]. Three major types of lamins, namely, A/C, B1 and B2 have been identified so far where B1 and B2 are expressed from two different genes *LMNB1* and *LMNB2* [18,19]. Lamin A & C are expressed from *LMNA* gene as splice variants. B-type lamins are expressed from the embryonic stage while A-type lamins are expressed in differentiated cells [20]. Lamin A is expressed as a premature protein pre-lamin A (664 amino acids) with a CAAX box which is farnesylated by farnesyltransferase and subsequently AAX is cleaved by an endopeptidase Rce1 (Ras-conversating enzyme 1) and/or Zmpste24 (Zinc metalloprotease related to Ste24p) to yield the mature lamin A having 18 fewer amino acid residues [21]. On the contrary, B-type lamins retain the isoprenylation thereby facilitating anchorage to the nuclear membrane. Like all other intermediate filaments (IFs), lamins also possess a characteristic tripartite motif consisting of an N-terminal head domain, an α-helical central coiled-coil forming rod domain and C-terminal domain which uniquely contains an immunoglobulin (Ig) domain [22]. *In vitro* analysis revealed that lamin assembly is initiated by dimer formation through the hydrophobic interaction in the helical regions and subsequently progress to form higher-order assembly via the parallel and head-to-tail and lateral compaction to form ~10 nm filaments [12,23]. However, this notion was also revised recently as the cryo-EM study revealed that lamins within nuclear lamina meshwork form tetramers of ~3.5 nm thick filament [24]. Lamins are differentially expressed among different tissue types, such as mechanically active tissues like muscle or cardiac tissue express more lamin A compared to the soft tissue like brain [25]. In retrospect, it has been shown earlier that lamin A is the principal mechanical component of the nucleus which attests to its preponderance in mechanically active tissues [26,27]. Several emerging reports suggest that external mechanical force is directly transmitted to the nucleus through the Linker of Nucleoskeleton and Cytoskeleton Complex (LINC) [28,29]. Integrin first senses the mechanical force from extracellular matrix (ECM) which is transmitted to actin and then subsequently propagated to the nucleoskeleton via the interaction with Nesprin 1/2, emerin, Sun protein which directly interact with nuclear lamins [30,31].

Over the past few decades, nearly 500 mutations have been identified in *LMNA* which cause a plethora of diseases such as Emery-Dreifuss Muscular Dystrophy (EDMD), dilated cardiomyopathy (DCM), Hutchinson-Gilford Progeria Syndrome (HGPS), Lipodystrophy syndrome, peripheral neuropathy, etc., which are collectively named as ‘laminopathies’[32]. The underlying reason behind these mutations and laminopathies still continues to elude the community of scientists. However, based on the discoveries of potential signaling pathways that go awry in laminopathies, researchers have shed some light that led to two different modes of explanation -the Structural and Gene regulation hypotheses [33]. The structural hypothesis suggests that lamin A mutations perturb higher-order assembly thereby leading to an aberrant network formation and result in a loss of nuclear shape and rigidity. As an effect, the link for external force transmission to the nucleus gets perturbed. Secondly, the gene regulation hypothesis suggests that lamin A participates in several nuclear processes like replication, transcription, splicing and epigenetic modification. Hence, lamin A mutations directly perturb those processes leading to aberrant gene expression. Nevertheless, these two hypotheses are not mutually exclusive but rather should be viewed as reconciliatory factors to explain the pathogenesis of laminopathies. In this perspective, we attempt to review a plausible mechanism to bring forth the role of other filaments like actin and their role in gene expression in tandem with lamin A.

## Importance of lamins inside the nucleoplasm and their role in organizing chromosomal contacts

**Figure 1.**
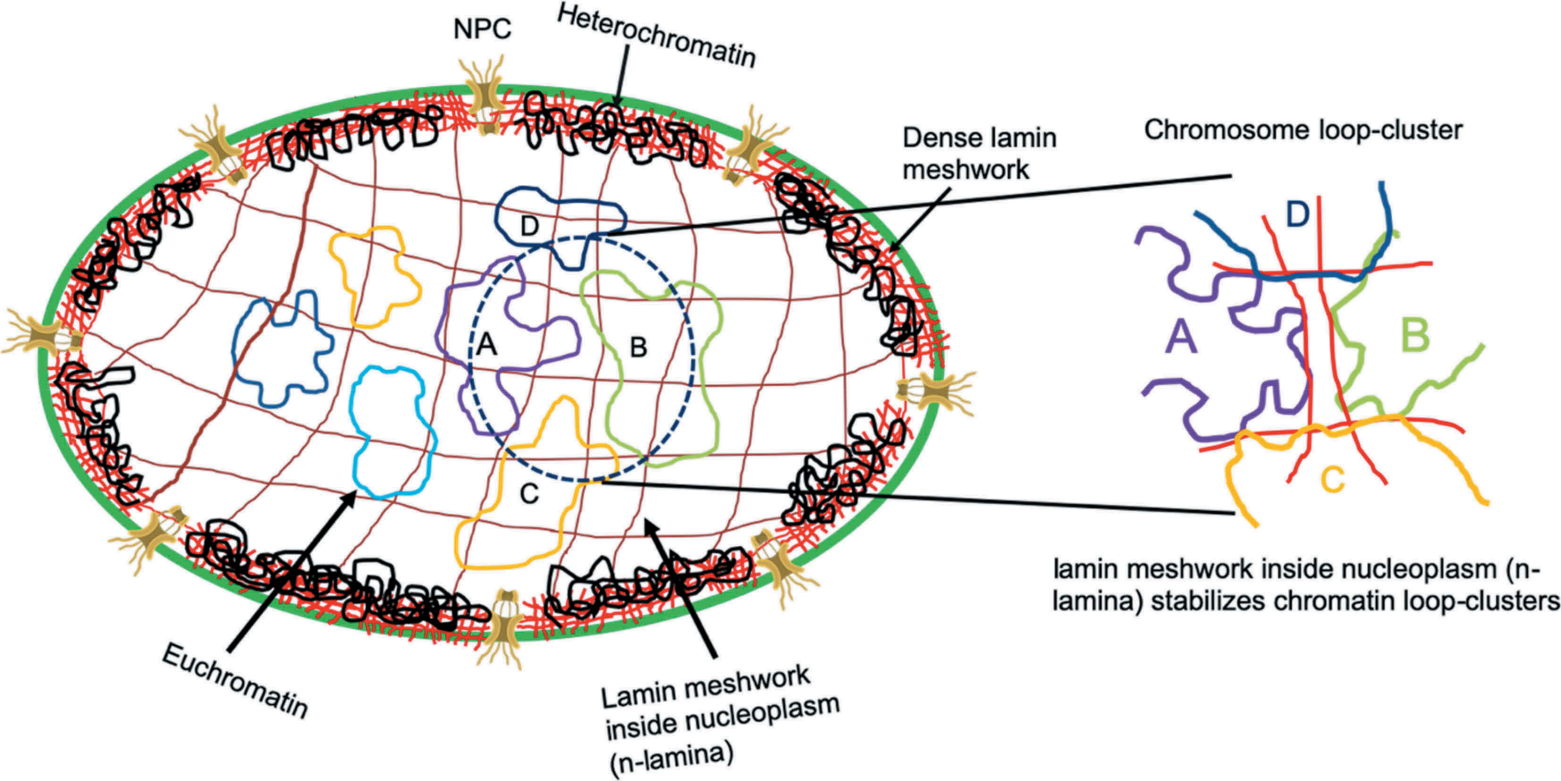
Schematic diagram of chromatin loop-cluster stabilization via lamin meshwork inside the nucleoplasm.

In prokaryotes, the nucleoid tethers to the plasma membrane to maintain its positioning [34]. Along the evolutionary pathway, eukaryotes evolved and acquired distinct nuclear boundaries to separate its chromosomal space from the cytoplasmic domain. All metazoans ranging from hydra to human consist of at least one form of lamins (B-type lamin) [35]. However, lower eukaryotes like yeast and *Arabidopsis thaliana* do not produce any lamin homologs [36]. In contrast to cytoplasmic IFs, the expression of at least one type of lamins is of paramount importance to maintain viability [37]. Sequence comparison studies also reflect that lamins are the most primitive IFs [38,39]. Most likely, essential nuclear membrane proteins like Nups (Nucleoporins), lamina associated polypeptides (LAP1) co-evolved with nuclear membrane [40]. With the evolution of higher eukaryotes, lamins also evolved and interactions with other nuclear envelope proteins help to organize chromatin architectures which may be indispensable for gene expression. It can be speculated that unlike cell membranes, the nuclear membrane does not face external mechanical fluctuations directly; therefore, the stiffness of the nuclear membrane need not be comparable to that of the plasma membrane but enough to retain its shape and rigidity. In addition, nuclei should be flexible enough to respond to the external force transmission as a feed-back mechanism to regulate gene expression. Hence, intermediate filaments emerged as the perfect candidate for these functions because of its unique mechanical stiffness property which is lower than both actin filaments and microtubules [41]. More importantly, persistence length is very small compared to the other filament systems which provide them with increased flexibility and bendability. Most biopolymers can be classified as semi-flexible as described by their bending rigidity according to the Worm-like chain (WLC) model [42]. Persistence length is the quantitative measurement of the stiffness of the polymers. The persistence lengths of double-stranded DNA, actin polymer, microtubule and IF are ~50 nm, ~10 μm, ~1 mm, 400–1000 nm, respectively, [43–46]. Therefore, microtubules are the stiffest polymer amongst all filamentous systems. Microtubules form mainly linear and rigid cables to facilitate active diffusion of the cargoes along their lengths as well as organelle movements [47]. Actin cables are strong enough to bear a load of organellar tethering, thereby ensuring the positional integrity of the organelles. Lamins amongst all other intermediate filaments are unique in a way because of their immunoglobulin (Ig) domains which interact with histones and other nuclear factors [48]. The major filament assembly happens through this long central helical domain which is also the principal load-bearing component of this protein [49]. The elastic nature of lamin A originates from long coiled-coil domain as it has been shown earlier [49]. The coiled-coil domains 1A, 1B, 2A and 2B are assembled around the heptad repeats and finally arranged into protofilament [12]. The disordered C-terminal domain also contains the Ig-fold topology which provides flexibility to the filament systems [50]. In the nucleoplasm, the chromosomes can assume specific positions through the interaction with the Ig domain [51,52]. Lamin protofilaments in *C. elegans* indicated remarkably high elasticity as persistence length was calculated to be ~167 nm [53]. *In vitro* assembled lamins and other IFs also exhibited persistence length to be 100–300 nm [54,55]. *In vitro*, microrheology experiments showed that lamin A network can resist up to 500% strain at 0.85 mg/ml concentration, while E161 K mutant could resist only up to >100%[56]. Several groups including ours have shown that in the presence of lamin A mutation, network assembly was severely perturbed and so was the elasticity [57–59]. Micropipette aspiration study has shown the elastic modulus to be ~25 mN/m [60]. Hence, lamin A meshwork acts as a mechanical shock absorber of nucleus and provides the major elastic contribution of nuclear envelope. However, lamins A and B also form a mesh-like structure inside the nucleoplasm apart from being present at the nuclear periphery [61,62]. Inside the nucleoplasm, lamin can exist in both soluble (phosphorylated) and insoluble meshwork (dephosphorylated) form. The ratio between the two forms can make lamin network very dynamic. Earlier, it had also been revealed that lamin A and lamin B form separate distinct but with some overlapping structure both in perinuclear and nucleoplasm [62]. Lamin B meshwork remains anchored predominantly to INM via the isoprenylation even during the mitosis [16]. Our group had showed previously that several mutants of lamin A bind to lamin B1 with differential stoichiometries and in these mutants, lamin B1 forms dilated meshwork [63]. Nevertheless, the influence on the lamin B network inside the nucleoplasm due to lamin A mutation is a little enigmatic. Henceforth, lamina at the nuclear periphery will be referred as peripheral lamina or p-lamina and lamin network in the nucleoplasm as nucleoplasmic lamina or n-lamina. Distribution of chromosomes inside nucleus is nonrandom; some of them are peripheral and some of them reside in the interior positions giving a characteristic heterochromatin and euchromatin distribution, respectively. The general notion is that peripheral chromosomes are heterochromatin which tends to be in closed conformations thereby inhibiting transcription as the accessibility of the other nuclear factors to this topology is rigorously limited. These heterochromatins are also tightly attached to the peripheral lamin meshwork. The other variety is euchromatin, which retains open conformation with ongoing active transcription. Most gene-poor chromosomes, which have less density of genes, tend to reside toward the periphery while gene-rich chromosomes maintain an interior distribution [64,65]. Chromosomes in open conformations often form loops which are maintained by the strong interactions of series of cohesin and CTCF molecules [66,67]. These loops are the hubs for transcriptions and other nuclear processes [68]. Although these are connected through cohesin molecules but still lack firm rigidity of their positioning. We propose that n-lamina provides the stability and rigidity for euchromatin positioning. Hence, the formation of the chromatin loop would also be tightly controlled by the n-lamina. Now, these loops often interact with the neighboring loop either from the same chromosome or in an inter-chromosomal fashion. To maintain consistent interactions, these loops require mechanical support. As depicted in Figure 1, p-lamina (in red color) forms a very dense network underlying the INM and remain strongly associated with the heterochromatin, whereas the n-lamina (in brown color) forms a dilated meshwork throughout the nucleoplasm where several euchromatin fibers are tethered. Often, the extension of these chromatin structures comes together (labeled as A, B, C and D) and form a loop-contact topology. N-lamina meshwork binds to the chromatin loops via histone and stabilizes that loop-contacts (zoomed image). So far, we have discussed the reason for the requirement of mechanical support systems inside the nucleoplasm in the context of chromatin loop stability. We will subsequently discuss the importance of these contacts in gene expression.

## Nuclear lamin and heterochromatin formation at the nuclear periphery

To better understand the relationship between nuclear lamin and gene expression, it is critical to delineate the role of lamin meshwork in chromatin packaging and their accessibility to the epigenetic and transcription factors. DNA adenine methyltransferase identification (DamID) method revealed the association between gene-poor or transcriptionally inactive heterochromatin to lamin B1 [69]. In general, chromosomes near the nuclear periphery are transcriptionally inactive but these chromatins can also modify their packaging occasionally and form the transcription loop structure [70,71]. Distribution of nuclear pore complexes (NPCs) on the surface is not random and the positionings of the NPCs and heterochromatin alternate with each other. NPCs are envisaged as gene gating components where the transcribing loop regions of the heterochromatin position themselves with the proximal NPCs and the transcripts would exit the nucleus through these NPCs [72]. The peripheral lamina can participate in the organization of these transcribing loops and help in the gene gating processes. The complete mechanism behind this is still unknown. The concentration of both lamin B1 and lamin A is quite high near the nuclear periphery; hence, it is quite intuitive that they might form a very dense meshwork [24]. As revealed recently, phase-separation of the nucleosome and corresponding histone modifications play a pivotal role in the heterochromatin formation [73,74]. We hypothesize that highly dense p-lamina creates a hydrogel-phase which helps in the heterochromatin formations and their maintenance. Thus, these factors, as well as lamin phosphorylation, can regulate this dense meshwork formation and subsequently lead to the stabilization of the heterochromatin. The concentration of lamins toward the interior region of the nucleus tapers off compared to the nuclear periphery [61,75,76]. We can presume that this lamin network is less dense compared to the periphery and there is a gel to sol transition from the periphery to the inner core. However, nucleoplasmic lamin meshwork or the n-lamina provides necessary structural stabilization of the inter-chromosomal contacts and topologically associating domains (TADs) formations. Like any other filamentous system, regulation of lamin assembly is directly controlled by phosphorylation [77]. These filaments are designed by evolution not only to provide mechanical support to chromosomes but also to facilitate the nuclear processes by melting locally and reversibly. The phosphorylation of lamins can provide such reversible dynamics based on the requirement of the chromosomes [78]. The local phosphorylation and dephosphorylation can help in movements of the loop contacts and formation of new contacts, respectively.

## Importance of the chromosomal contacts

Eukaryotic, specifically mammalian genomes are organized into two conformations, active and inactive compartments. Recent findings suggest that each chromosome consists of several distinct domains, called TADs [79,80]. These TADs can be 100 k base pairs to several megabase pairs long and these are stably maintained throughout the cell cycle and evolutionarily conserved in related species [80,81]. The chromatin loop extrusion model elucidates the organization of these TADs and explains the TAD formations through the interaction of the ring-shaped cohesin and transcription factor CTCF proteins [82]. An overwhelming amount of literature suggests that the chromosome often forms loops that are maintained by a series of cohesin molecules [83,84]. Now, these loops regulate the gene expression forming open conformations allowing nuclear factors to interact [85]. This loop formation also provides long-range interaction where the distant promoter can physically come closer to control the gene expression [86]. Especially in immune cells, where the gene clustering is a common phenomenon to regulate certain processes [87]; several genes either from same chromosome or from different chromosomes come together to form a cluster which can be regulated in an isometric manner by the same promoter [88]. These chromosomal contacts are very cell-specific and depend on the cell cycle stage. Although evidence for chromosomal contacts is very promising, how these contacts regulate the gene expression and subsequent cellular function is yet to be uncovered. Earlier, we had hypothesized how these contacts can regulate functions [89]. During the transition from prokaryotic genome to eukaryotes, eukaryotes had lost the polycistronic nature of the genome and functionally relevant genes are distributed throughout genomes, often in different chromosomes. It’s questionable whether these chromosomal contacts are reminiscent of the polycistronic nature of prokaryotes. However, these contacts directly affect the gene-expression profile of the cell. As chromosomes interact directly with the lamina meshwork, it can be hypothesized that the lamina helps to position and maintain these chromosomal contacts. In addition, the filamentous nature of lamins directly controls the stability of inter/intrachromosomal loops and their long-range interactions, thereby regulating gene expression and their subsequent functions.

## Positioning matters: neighborhood controls the efficacy of gene expressions

An emerging concept of ‘phase-separation’ dominates several nuclear processes like transcription, splicing, etc. Phase-separation provides a local compartment to execute these processes where local concentration of individual components can be reliably maintained. As mentioned earlier, several chromosomes come together and form a phase-separated loop cluster [90]. These phase-separated condensates can regulate transcription and splicing in the cohort. In the early 1980 s, Cremer laboratory first showed that each chromosome maintains its specific location during interphase [91]. Subsequently, it has also been shown that gene-rich chromosomes (such as Ch 19) tend to be in the interior region of the nucleus while gene-poor chromosomes (such as Ch 18) reside in the peripheral region [65]. Emerging Hi-C techniques arguably suggest that each individual chromosome possesses a preferential location and neighborhood inside the nucleus. Hence, maintaining the neighborhood may be important to regulate gene expression. Earlier, it had been proposed that these chromosomal contacts or loop clusters can increase the efficiency and efficacy in the protein complex formation [89]. Nevertheless, through long-range interactions and gene cluster formation, a single promoter can regulate gene expression [92,93]. Therefore, stable maintenance of these clusters and their precise positioning account for their expression and subsequent functions. It is thus proposed that the n-lamina maintains and positions these phase-separated clusters. Although n-lamina may be dispensable to initiate the chromosomal contacts and gene clusters formation, nonetheless, lamin can provide the mechanical support to stabilize these clusters.

The most important question is why neighborhood matters for chromosomes. Chromosomes are long stretchable strings that are compacted by multiple orders of packaging. These open up locally in an open conformation. Now unlike a small molecule, a single chromosome can participate in multiple loop formations in multiple places simultaneously; therefore, their mechanical rigidity is very important to maintain these loops and loop clusters with other chromosomes. The probability of multiple loop formation with multiple neighbors would be very less and stochastically controlled until their positions are very tightly regulated. Moreover, as the chromosome needs to be stretched to form loop-clusters, we hypothesize that small actin cable and myosin motors provide the active energy and route to facilitate this phenomenon.

## Small actin cables dictate movements of chromosomal contacts through active diffusion

**Figure 2.**
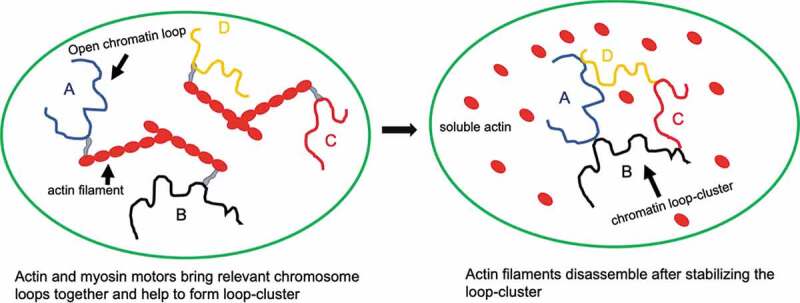
Active movements of chromatin loops inside the nucleoplasm.

There has been a longstanding debate regarding the presence of actin and myosin motors inside the nucleoplasm. However, it has been established arguably that actin filaments do exist inside the nucleus along with myosin motors [9]. Here, we will attempt to provide some insights and subsequently a hypothesis to support the requirement of actin cables. As we mentioned earlier, chromatin positioning varies depending on the cell types and cell cycles. However, for consistent gene-expression patterns, specific loops-clusters formations are indispensable. We propose that these loop-clusters are established by the actin cable with the help of myosin motors. Specific cognate chromatin organization elements may bind to myosin and actin cable and loops-clusters can be established via the interaction of the other receptor binding elements. Nuclear actins do not necessarily form long cables like the cytosolic form, but, form short cables which are highly dynamic in nature [94]. Now, the pertinent question may arise – what is the requirement of these cables inside the nucleus when lamin meshwork is already existent? These actin cables may not be required for the mechanical support inside the nucleoplasm, but unlike lamin filaments, actin filaments can participate as a vehicle of active diffusion [95]. Therefore, it can be argued that actin and myosin mediated active diffusion can facilitate transient chromosomal contact formation (either intra or inter) inside the nucleoplasm. For inter-chromosomal contact formations, the individual chromosome opens and forms a stable loop through the help of cohesin molecules and multiple loops eventually come together via the active diffusion mediated by the small actin cables and myosin motors and subsequently, these loop clusters are mechanically anchored and stabilized by the lamin filaments. This hypothesis has been illustrated in Figure 2 which is a cartoon to explain how four chromatin loops A, B, C and D are initially located distantly and so form a loop-cluster among themselves just following Brownian diffusion which may take very long time considering a slow diffusion rate of the chromosome inside nucleoplasm. Histones and other chromatin packaging proteins can bind to actin and myosin [96–98]; therefore, these loops can diffuse easily on the actin cable using myosin motor proteins. Now, to form A-B-C-D loop-clusters consistently, there must be cognate binding partners which can recognize their partners. These cognate partners may uniquely bind to the sequence of individual loop. The small actin filaments have enough persistent length to provide the flexibility and direct chromosomal transition from one place to another. These filaments are very dynamic and transient in nature. Therefore, once active loop clusters are firmly established, these filaments can be disassembled through phosphorylation. The high bendability and small diameter enable these filaments to pass through the thicker lamina meshwork without disassembling it. However, there is no experimental evidence yet to reinforce this hypothesis and it may very well be refutable.

## Perturbation in filaments and aberrant gene expression

Numerous examples have shown that mutations in the lamin A protein lead to several diseases commonly called laminopathies [99,100]. Previously, it had been shown that in DCM and EDMD cells, there was significant inhibition in myoblast differentiation [101] and downregulation of MyoD expression [102]. The pluripotent stem cell consisting DCM causing *LMNA* mutation produced a elevated stress-responsive MEK1/ERK1/2 pathway leading to apoptosis [103]. Lipodystrophic lamin A R482 W mutation perturbs chromatin conformation at an anti-adipogenic microRNA locus in adipose progenitor cells and EDMD causing R453 W mutant alters the lamin-associated domains (LADs) and lamin-Polycomb repressor complex interactions [104]. Muscular dystrophic patients showed a severe discrepancy in the Micro-RNA expression profile [105]. It had been established earlier that due to these mutations, mechanical stability even at dimer level and higher-order assembly had been abrogated which subsequently depleted the strain bearing capacity [49,56]. Often due to these mutations, lamin A forms aggregates inside the nucleoplasm [106]. Now, one could challenge how lamin A mutation can alter gene expression. Lamin A interacts with lamin-associated domains (LADs) that are scattered throughout the genome and participate in epigenetic modification and regulation of gene expression [104]. Hence, defects in the lamin A assembly affect the lamin A matrix underlying the inner nuclear membrane and the meshwork inside the nucleoplasm. As a result, the mechanical rigidity of the INM gets sacrificed and nuclei become vulnerable to any mechanical stress. As the rigidity of the nuclear membrane also goes down, under any stressed condition, chromosomes attached to the nuclear membrane and loop protrusions from these chromosomes would be directly affected, hence, it would lead to altered gene expression. According to our proposed hypothesis, n-lamina distribution can be severely impaired due to *LMNA* mutation; hence, the stability of chromosome loop-clusters can be severely scarified. N-lamina can no longer provide the mechanical support for any chromosomal contacts, gene clusters and long-range interactions in chromosomes become unstable. As a result, gene expression patterns from these clusters can be altered. In addition, depending on the loss of these loop-cluster formations, different cell types can produce different phenotypes because of the same lamin A. mutation. Earlier, hypotheses could not answer the reasoning behind different disease phenotypes in different cell types. However, our hypothesis can predict the possibility of the outcome if one could identify the loss of the chromosomal contacts. In addition, mutations in actin and myosin proteins can also produce similar effects.

## Conclusion and perspective

Rabl proposed the territorial organization of the chromosome in 1885 [107] and a century later since Cremer et al. elucidated that each chromosome exists as distinct territory inside the nucleus [91], the genome architecture and their organizations have drawn considerable attention of many scientists. However, it is still an enigma to provide a holistic explanation of how chromosome organizes themselves and regulate their gene expression. Chromosomes consist of several TADs and chromatin in open conformation form loops which are transcriptionally active. Although lamins form a dense meshwork underlying the INM and provide structural rigidity to the nucleus, lamins also control chromatin dynamics and gene expression. In this article, we have attempted to present a new perspective on how lamin can regulate gene expression. Briefly, lamins form less dense meshwork inside the nucleoplasm which stably maintain the inter- and intra-chromosomal contacts. These chromosomal contacts can form a phase-separated cluster where transcription and their expression can be regulated in a concerted manner. These contacts are not random but active diffusion through the interactions with small actin cables and myosin motors ensures these contact formations and provide dynamicity. However, due to lamin A mutation, there could be a perturbation in lamin assembly thereby leading to an alteration in the stability of chromosomal contacts or loop-clusters. Nature of the alterations in the chromosomal contacts can dictate the aberrant gene-expression pattern and as a result, different cell types can produce different phenotypes because of the similar lamin A mutations. This hypothesis, therefore, can answer the puzzle behind lamin A mutations and laminopathies in different tissue types. Even though, B-type lamins are indispensable for cell viability, whether the perturbation in the chromosomal contacts inside nucleoplasm due to lamin A mutations can be rescued by membrane-anchored lamin B is questionable. Although an overwhelming amount of literature on lamin A mutations and laminopathies exists, still a systematic investigation involving lamin A network formation and its interaction with chromosomal contacts or loop-clusters subsequently modulating gene expressions is needed.

## References

[1] Schnitzer MJ, Visscher K, Block SM. Force production by single kinesin motors. Nat Cell Biol. 2000;2(10):718–723. 11025662 10.1038/35036345

[2] Wickstead B, Gull K. The evolution of the cytoskeleton. J Cell Biol. 2011;194(4):513–525. 21859859 10.1083/jcb.201102065 PMC3160578

[3] Zimek A, Stick R, Weber K. Genes coding for intermediate filament proteins: common features and unexpected differences in the genomes of humans and the teleost fish fugu rubripes. J Cell Sci. 2003;116(11):2295–2302. 12697841 10.1242/jcs.00444

[4] Blumenberg M. Evolution of homologous domains of cytoplasmic intermediate filament proteins and lamins. Mol Biol Evol. 1989;6(1):53–65. 2921943 10.1093/oxfordjournals.molbev.a040533

[5] Herrmann H, Aebi U. Intermediate filaments: structure and assembly. Cold Spring Harb Perspect Biol. 2016;8(11):a018242. 10.1101/cshperspect.a018242 PMC5088526 27803112

[6] Gerace L, Huber MD. Nuclear lamina at the crossroads of the cytoplasm and nucleus. J Struct Biol. 2012;177(1):24–31. 22126840 10.1016/j.jsb.2011.11.007 PMC3261324

[7] Dahl KN, Ribeiro AJ, Lammerding J. Nuclear shape, mechanics, and mechanotransduction. Circ Res. 2008;102(11):1307–1318. 18535268 10.1161/CIRCRESAHA.108.173989 PMC2717705

[8] de Lanerolle P, Serebryannyy L. Nuclear actin and myosins: life without filaments. Nat Cell Biol. 2011;13(11):1282–1288. 22048410 10.1038/ncb2364

[9] de Lanerolle P. Nuclear actin and myosins at a glance. J Cell Sci. 2012;125(21):4945–4949. 23277533 10.1242/jcs.099754 PMC3533385

[10] Petry S. Mechanisms of mitotic spindle assembly. Annu Rev Biochem. 2016;85(1):659–683. 27145846 10.1146/annurev-biochem-060815-014528 PMC5016079

[11] Goldman RD, Gruenbaum Y, Moir RD, et al. Nuclear lamins: building blocks of nuclear architecture. Genes Dev. 2002;16(5):533–547. 11877373 10.1101/gad.960502

[12] Stuurman N, Heins S, Aebi U. Nuclear lamins: their structure, assembly, and interactions. J Struct Biol. 1998;122(1–2):42–66. 9724605 10.1006/jsbi.1998.3987

[13] Dialynas G, Speese S, Budnik V, et al. The role of drosophila lamin C in muscle function and gene expression. Development. 2010;137(18):3067–3077. 20702563 10.1242/dev.048231 PMC2926956

[14] Aebi U, Cohn J, Buhle L, et al. The nuclear lamina is a meshwork of intermediate-type filaments. Nature. 1986;323(6088):560–564. 3762708 10.1038/323560a0

[15] Fawcett DW. On the occurrence of a fibrous lamina on the inner aspect of the nuclear envelope in certain cells of vertebrates. Am J Anat. 1966;119(1):129–145. 6007824 10.1002/aja.1001190108

[16] Gerace L, Blobel G. The nuclear envelope lamina is reversibly depolymerized during mitosis. Cell. 1980;19(1):277–287. 7357605 10.1016/0092-8674(80)90409-2

[17] Gerace L, Blum A, Blobel G. Immunocytochemical localization of the major polypeptides of the nuclear pore complex-lamina fraction. Interphase and mitotic distribution. J Cell Biol. 1978;79(2):546–566. 102651 10.1083/jcb.79.2.546 PMC2110258

[18] Schumacher J, Reichenzeller M, Kempf T, et al. Identification of a novel, highly variable amino-terminal amino acid sequence element in the nuclear intermediate filament protein lamin B(2) from higher vertebrates. FEBS Lett. 2006;580(26):6211–6216. 17070523 10.1016/j.febslet.2006.10.023

[19] Broers JL, Machiels BM, Kuijpers HJH, *et al*. A- and B-type lamins are differentially expressed in normal human tissues. Histochem Cell Biol. 1997;107(6):505–517. 9243284 10.1007/s004180050138

[20] Kim Y, Sharov AA, McDole K, *et al*. Mouse B-type lamins are required for proper organogenesis but not by embryonic stem cells. Science. 2011;334(6063):1706–1710. 22116031 10.1126/science.1211222 PMC3306219

[21] Corrigan DP, KUSZCZAK D, RUSINOL A, *et al*. Prelamin A endoproteolytic processing in vitro by recombinant Zmpste24. Biochem J. 2005;387(1):129–138. 15479156 10.1042/BJ20041359 PMC1134940

[22] Herrmann H, Aebi U. Intermediate filaments: molecular structure, assembly mechanism, and integration into functionally distinct intracellular scaffolds. Annu Rev Biochem. 2004;73(1):749–789. 15189158 10.1146/annurev.biochem.73.011303.073823

[23] Heitlinger E, Peter M, Häner M, *et al*. Expression of chicken lamin B2 in escherichia coli: characterization of its structure, assembly, and molecular interactions. J Cell Biol. 1991;113(3):485–495. 2016332 10.1083/jcb.113.3.485 PMC2288961

[24] Turgay Y, Eibauer M, Goldman AE, *et al*. The molecular architecture of lamins in somatic cells. Nature. 2017;543(7644):261–264. 28241138 10.1038/nature21382 PMC5616216

[25] Swift J, Ivanovska IL, Buxboim A, *et al*. Nuclear lamin-A scales with tissue stiffness and enhances matrix-directed differentiation. Science. 2013;341(6149):1240104. 23990565 10.1126/science.1240104 PMC3976548

[26] Lammerding J, Fong LG, Ji JY, *et al*. Lamins A and C but not lamin B1 regulate nuclear mechanics. J Biol Chem. 2006;281(35):25768–25780. 16825190 10.1074/jbc.M513511200

[27] Lele TP, Dickinson RB, Gundersen GG. Mechanical principles of nuclear shaping and positioning. J Cell Biol. 2018;217(10):3330–3342. 30194270 10.1083/jcb.201804052 PMC6168261

[28] Lombardi ML, Lammerding J. Keeping the LINC: the importance of nucleocytoskeletal coupling in intracellular force transmission and cellular function. Biochem Soc Trans. 2011;39(6):1729–1734. 22103516 10.1042/BST20110686 PMC4589539

[29] Hao H, Starr DA. SUN/KASH interactions facilitate force transmission across the nuclear envelope. Nucleus. 2019;10(1):73–80. 30888237 10.1080/19491034.2019.1595313 PMC6527376

[30] Sun Z, Guo SS, Fassler R. Integrin-mediated mechanotransduction. J Cell Biol. 2016;215(4):445–456. 27872252 10.1083/jcb.201609037 PMC5119943

[31] Lombardi ML, Jaalouk, D. E., Shanahan, C. M *et al*. The interaction between nesprins and sun proteins at the nuclear envelope is critical for force transmission between the nucleus and cytoskeleton. J Biol Chem. 2011;286(30):26743–26753. 21652697 10.1074/jbc.M111.233700 PMC3143636

[32] Worman HJ, Bonne G. Laminopathies”: a wide spectrum of human diseases. Exp Cell Res. 2007;313(10):2121–2133. 17467691 10.1016/j.yexcr.2007.03.028 PMC2964355

[33] Osmanagic-Myers S, Foisner R. The structural and gene expression hypotheses in laminopathic diseases-not so different after all. Mol Biol Cell. 2019;30(15):1786–1790. 31306095 10.1091/mbc.E18-10-0672 PMC6727745

[34] Magnan D, Joshi MC, Barker AK, et al. DNA replication initiation is blocked by a distant chromosome-membrane attachment. Curr Biol. 2015;25(16):2143–2149. 26255849 10.1016/j.cub.2015.06.058 PMC4546506

[35] Erber A, Riemer D, Hofemeister H, *et al*. Characterization of the hydra lamin and its gene: A molecular phylogeny of metazoan lamins. J Mol Evol. 1999;49(2):260–271. 10441677 10.1007/pl00006548

[36] Dittmer TA, Misteli T. The lamin protein family. Genome Biol. 2011;12(5):222. 21639948 10.1186/gb-2011-12-5-222 PMC3219962

[37] Harborth J, Elbashir SM, Bechert K, et al. Identification of essential genes in cultured mammalian cells using small interfering RNAs. J Cell Sci. 2001;114(Pt 24):4557–4565. 11792820 10.1242/jcs.114.24.4557

[38] Riemer D, Weber K. Common and variant properties of intermediate filament proteins from lower chordates and vertebrates; two proteins from the tunicate styela and the identification of a type III homologue. J Cell Sci. 1998;111(Pt 19):2967–2975. 9730988 10.1242/jcs.111.19.2967

[39] Dodemont H, Riemer D, Weber K. Structure of an invertebrate gene encoding cytoplasmic intermediate filament (IF) proteins: implications for the origin and the diversification of IF proteins. Embo J. 1990;9(12):4083–4094. 2249666 10.1002/j.1460-2075.1990.tb07630.x PMC552181

[40] Mans BJ, Anantharaman V, Aravind L, et al. Comparative genomics, evolution and origins of the nuclear envelope and nuclear pore complex. Cell Cycle. 2004;3(12):1612–1637. 15611647 10.4161/cc.3.12.1316

[41] Pegoraro AF, Janmey P, Weitz DA. Mechanical properties of the cytoskeleton and cells. Cold Spring Harb Perspect Biol. 2017;9(11):a022038. 10.1101/cshperspect.a022038 PMC5666633 29092896

[42] Marko JF, Siggia ED. Statistical mechanics of supercoiled DNA. Phys Rev E Stat Phys Plasmas Fluids Relat Interdiscip Topics. 1995;52(3):2912–2938. 9963738 10.1103/physreve.52.2912

[43] McCauley MJ, Williams MC. Mechanisms of DNA binding determined in optical tweezers experiments. Biopolymers. 2007;85(2):154–168. 17080421 10.1002/bip.20622

[44] van Mameren J, Vermeulen KC, Gittes F, et al. Leveraging single protein polymers to measure flexural rigidity. J Phys Chem B. 2009;113(12):3837–3844. 19673071 10.1021/jp808328a

[45] Wen Q, Janmey PA. Polymer physics of the cytoskeleton. Curr Opin Solid State Mater Sci. 2011;15(5):177–182. 22081758 10.1016/j.cossms.2011.05.002 PMC3210450

[46] Block J, Schroeder V, Pawelzyk P, et al. Physical properties of cytoplasmic intermediate filaments. Biochim Biophys Acta. 2015;1853(11):3053–3064. 25975455 10.1016/j.bbamcr.2015.05.009

[47] Barlan K, Gelfand VI. Microtubule-based transport and the distribution, tethering, and organization of organelles. Cold Spring Harb Perspect Biol. 2017;9(5):a025817. 10.1101/cshperspect.a025817 PMC5411697 28461574

[48] Dechat T, Adam SA, Goldman RD. Nuclear lamins and chromatin: when structure meets function. Adv Enzyme Regul. 2009;49(1):157–166. 19154754 10.1016/j.advenzreg.2008.12.003 PMC3253622

[49] Bera M, Ainavarapu SR, Sengupta K. Significance of 1B and 2B domains in modulating elastic properties of lamin A. Sci Rep. 2016;6(1):27879. 27301336 10.1038/srep27879 PMC4908593

[50] Bera M, Kotamarthi HC, Dutta S, *et al*. Characterization of unfolding mechanism of human lamin A Ig fold by single-molecule force spectroscopy-implications in EDMD. Biochemistry. 2014;53(46):7247–7258. 25343322 10.1021/bi500726f

[51] Taniura H, Glass C, Gerace L. A chromatin binding site in the tail domain of nuclear lamins that interacts with core histones. J Cell Biol. 1995;131(1):33–44. 7559784 10.1083/jcb.131.1.33 PMC2120604

[52] Bruston F, Delbarre E, Östlund C, *et al*. Loss of a DNA binding site within the tail of prelamin A contributes to altered heterochromatin anchorage by progerin. FEBS Lett. 2010;584(14):2999–3004. 20580717 10.1016/j.febslet.2010.05.032 PMC2908524

[53] Grossman E, Dahan I, Stick R, *et al*. Filaments assembly of ectopically expressed caenorhabditis elegans lamin within xenopus oocytes. J Struct Biol. 2012;177(1):113–118. 22085746 10.1016/j.jsb.2011.11.002

[54] Mucke N, Kreplak, L., Kirmse, R *et al*. Assessing the flexibility of intermediate filaments by atomic force microscopy. J Mol Biol. 2004;335(5):1241–1250. 14729340 10.1016/j.jmb.2003.11.038

[55] Kreplak L, Herrmann H, Aebi U. Tensile properties of single desmin intermediate filaments. Biophys J. 2008;94(7):2790–2799. 18178641 10.1529/biophysj.107.119826 PMC2267133

[56] Banerjee A, Rathee V, Krishnaswamy R, *et al*. Viscoelastic behavior of human lamin A proteins in the context of dilated cardiomyopathy. PLoS One. 2013;8(12):e83410. 24386194 10.1371/journal.pone.0083410 PMC3875444

[57] Buxboim A, Irianto J, Swift J, *et al*. Coordinated increase of nuclear tension and lamin-A with matrix stiffness outcompetes lamin-B receptor that favors soft tissue phenotypes. Mol Biol Cell. 2017;28(23):3333–3348. 28931598 10.1091/mbc.E17-06-0393 PMC5687034

[58] van Tienen FHJ, Lindsey PJ, Kamps MAF, *et al*. Assessment of fibroblast nuclear morphology aids interpretation of LMNA variants. Eur J Hum Genet. 2019;27(3):389–399. 30420677 10.1038/s41431-018-0294-0 PMC6460565

[59] Laurini E, Martinelli, V., Lanzicher, T.*et al*. Biomechanical defects and rescue of cardiomyocytes expressing pathologic nuclear lamins. Cardiovasc Res. 2018;114(6):846–857. 29432544 10.1093/cvr/cvy040 PMC5909658

[60] Dahl KN, Kahn SM, Wilson KL, et al. The nuclear envelope lamina network has elasticity and a compressibility limit suggestive of a molecular shock absorber. J Cell Sci. 2004;117(20):4779–4786. 15331638 10.1242/jcs.01357

[61] Xie W, Chojnowski A, Boudier T, *et al*. A-type lamins form distinct filamentous networks with differential nuclear pore complex associations. Curr Biol. 2016;26(19):2651–2658. 27641764 10.1016/j.cub.2016.07.049

[62] Shimi T, Pfleghaar, K., Kojima, S. I. *et al*. The A- and B-type nuclear lamin networks: microdomains involved in chromatin organization and transcription. Genes Dev. 2008;22(24):3409–3421. 19141474 10.1101/gad.1735208 PMC2607069

[63] Bhattacharjee P, Dasgupta D, Sengupta K. DCM associated LMNA mutations cause distortions in lamina structure and assembly. Biochim Biophys Acta Gen Subj. 2017;1861(11):2598–2608. 28844980 10.1016/j.bbagen.2017.08.016

[64] Bickmore WA. The spatial organization of the human genome. Annu Rev Genomics Hum Genet. 2013;14(1):67–84. 23875797 10.1146/annurev-genom-091212-153515

[65] Cremer T, Cremer C. Chromosome territories, nuclear architecture and gene regulation in mammalian cells. Nat Rev Genet. 2001;2(4):292–301. 11283701 10.1038/35066075

[66] de Wit E, Vos EM, Holwerda SB, *et al*. CTCF binding polarity determines chromatin looping. Mol Cell. 2015;60(4):676–684. 26527277 10.1016/j.molcel.2015.09.023

[67] Hansen AS, Pustova I, Cattoglio C, et al. CTCF and cohesin regulate chromatin loop stability with distinct dynamics. Elife. 2017;6. DOI:10.7554/eLife.25776. PMC5446243 28467304

[68] Kadauke S, Blobel GA. Chromatin loops in gene regulation. Biochim Biophys Acta. 2009;1789(1):17–25. 18675948 10.1016/j.bbagrm.2008.07.002 PMC2638769

[69] van Steensel B, Belmont AS. Lamina-associated domains: links with chromosome architecture, heterochromatin, and gene repression. Cell. 2017;169(5):780–791. 28525751 10.1016/j.cell.2017.04.022 PMC5532494

[70] Guelen L, Pagie L, Brasset E, *et al*. Domain organization of human chromosomes revealed by mapping of nuclear lamina interactions. Nature. 2008;453(7197):948–951. 18463634 10.1038/nature06947

[71] Saksouk N, Simboeck E, Dejardin J. Constitutive heterochromatin formation and transcription in mammals. Epigenetics Chromatin. 2015;8(1):3. 25788984 10.1186/1756-8935-8-3 PMC4363358

[72] Blobel G. Gene gating: a hypothesis. Proc Natl Acad Sci U S A. 1985;82(24):8527–8529. 3866238 10.1073/pnas.82.24.8527 PMC390949

[73] Sanulli S, Trnka MJ, Dharmarajan V, *et al*. HP1 reshapes nucleosome core to promote phase separation of heterochromatin. Nature. 2019;575(7782):390–394. 31618757 10.1038/s41586-019-1669-2 PMC7039410

[74] Strom AR, Emelyanov AV, Mir M, *et al*. Phase separation drives heterochromatin domain formation. Nature. 2017;547(7662):241–245. 28636597 10.1038/nature22989 PMC6022742

[75] Goldberg MW, Huttenlauch I, Hutchison CJ, et al. Filaments made from A- and B-type lamins differ in structure and organization. J Cell Sci. 2008;121(2):215–225. 18187453 10.1242/jcs.022020

[76] Shimi T, Kittisopikul M, Tran J, *et al*. Structural organization of nuclear lamins A, C, B1, and B2 revealed by superresolution microscopy. Mol Biol Cell. 2015;26(22):4075–4086. 26310440 10.1091/mbc.E15-07-0461 PMC4710238

[77] Torvaldson E, Kochin V, Eriksson JE. Phosphorylation of lamins determine their structural properties and signaling functions. Nucleus. 2015;6(3):166–171. 25793944 10.1080/19491034.2015.1017167 PMC4615644

[78] Foisner R, Gerace L. Integral membrane proteins of the nuclear envelope interact with lamins and chromosomes, and binding is modulated by mitotic phosphorylation. Cell. 1993;73(7):1267–1279. 8324822 10.1016/0092-8674(93)90355-t

[79] Dixon JR, Selvaraj S, Yue F, *et al*. Topological domains in mammalian genomes identified by analysis of chromatin interactions. Nature. 2012;485(7398):376–380. 22495300 10.1038/nature11082 PMC3356448

[80] Dixon JR, Gorkin DU, Ren B. Chromatin domains: the unit of chromosome organization. Mol Cell. 2016;62(5):668–680. 27259200 10.1016/j.molcel.2016.05.018 PMC5371509

[81] Dekker J, Heard E. Structural and functional diversity of topologically associating domains. FEBS Lett. 2015;589(20PartA):2877–2884. 26348399 10.1016/j.febslet.2015.08.044 PMC4598308

[82] Nasmyth K. Disseminating the genome: joining, resolving, and separating sister chromatids during mitosis and meiosis. Annu Rev Genet. 2001;35(1):673–745. 11700297 10.1146/annurev.genet.35.102401.091334

[83] Guillou E, Ibarra A, Coulon V, *et al*. Cohesin organizes chromatin loops at DNA replication factories. Genes Dev. 2010;24(24):2812–2822. 21159821 10.1101/gad.608210 PMC3003199

[84] Wutz G, Várnai C, Nagasaka K, *et al*. Topologically associating domains and chromatin loops depend on cohesin and are regulated by CTCF, WAPL, and PDS5 proteins. Embo J. 2017;36(24):3573–3599. 29217591 10.15252/embj.201798004 PMC5730888

[85] Holwerda S, de Laat W. Chromatin loops, gene positioning, and gene expression. Front Genet. 2012;3:217. 23087710 10.3389/fgene.2012.00217 PMC3473233

[86] Dekker J, Misteli T. Long-range chromatin interactions. Cold Spring Harb Perspect Biol. 2015;7(10):a019356. 26430217 10.1101/cshperspect.a019356 PMC4588061

[87] Makino T, McLysaght A. Interacting gene clusters and the evolution of the vertebrate immune system. Mol Biol Evol. 2008;25(9):1855–1862. 18573844 10.1093/molbev/msn137

[88] Chambeyron S, Bickmore WA. Does looping and clustering in the nucleus regulate gene expression? Curr Opin Cell Biol. 2004;16(3):256–262. 15145349 10.1016/j.ceb.2004.03.004

[89] Bera M, Kalyana Sundaram RV. Chromosome territorial organization drives efficient protein complex formation: a hypothesis. Yale J Biol Med. 2019;92(3):541–548. 31543715 PMC6747946

[90] Hnisz D, Shrinivas K, Young RA, et al. A phase separation model for transcriptional control. Cell. 2017;169(1):13–23. 28340338 10.1016/j.cell.2017.02.007 PMC5432200

[91] Cremer T, Cremer, C., Baumann, H. *et al*. Rabl’s model of the interphase chromosome arrangement tested in Chinese hamster cells by premature chromosome condensation and laser-UV-microbeam experiments. Hum Genet. 1982;60(1):46–56. 7076247 10.1007/BF00281263

[92] Sanyal A, Lajoie BR, Jain G, et al. The long-range interaction landscape of gene promoters. Nature. 2012;489(7414):109–113. 22955621 10.1038/nature11279 PMC3555147

[93] Bartkuhn M, Renkawitz R. Long range chromatin interactions involved in gene regulation. Biochim Biophys Acta. 2008;1783(11):2161–2166. 18706938 10.1016/j.bbamcr.2008.07.011

[94] Spichal M, Brion A, Herbert S, *et al*. Evidence for a dual role of actin in regulating chromosome organization and dynamics in yeast. J Cell Sci. 2016;129(4):681–692. 26763908 10.1242/jcs.175745

[95] Walker ML, Burgess SA, Sellers JR, *et al*. Two-headed binding of a processive myosin to F-actin. Nature. 2000;405(6788):804–807. 10866203 10.1038/35015592

[96] Hurst V, Shimada K, Gasser SM. Nuclear actin and actin-binding proteins in DNA repair. Trends Cell Biol. 2019;29(6):462–476. 30954333 10.1016/j.tcb.2019.02.010

[97] Kelpsch DJ, Tootle TL. Nuclear actin: from discovery to function. Anat Rec (Hoboken). 2018;301(12):1999–2013. 30312531 10.1002/ar.23959 PMC6289869

[98] Klages-Mundt NL, Kumar A, Zhang Y, et al. The nature of actin-family proteins in chromatin-modifying complexes. Front Genet. 2018;9:398. 30319687 10.3389/fgene.2018.00398 PMC6167448

[99] Broers JL, Hutchison CJ, Ramaekers FC. Laminopathies. J Pathol. 2004;204(4):478–488. 15495262 10.1002/path.1655

[100] Capell BC, Collins FS. Human laminopathies: nuclei gone genetically awry. Nat Rev Genet. 2006;7(12):940–952. 17139325 10.1038/nrg1906

[101] Favreau C, Higuet D, Courvalin JC, et al. Expression of a mutant lamin A that causes emery-dreifuss muscular dystrophy inhibits in vitro differentiation of C2C12 myoblasts. Mol Cell Biol. 2004;24(4):1481–1492. 14749366 10.1128/MCB.24.4.1481-1492.2004 PMC344177

[102] Frock RL, Kudlow, B. A., Evans, A. M *et al*. Lamin A/C and emerin are critical for skeletal muscle satellite cell differentiation. Genes Dev. 2006;20(4):486–500. 16481476 10.1101/gad.1364906 PMC1369050

[103] Siu C-W, Lee Y-K, Ho JC-Y, *et al*. Modeling of lamin A/C mutation premature cardiac aging using patient-specific induced pluripotent stem cells. Aging (Albany NY). 2012;4(11):803–822. 23362510 10.18632/aging.100503 PMC3560431

[104] Briand N, Collas P. Laminopathy-causing lamin A mutations reconfigure lamina-associated domains and local spatial chromatin conformation. Nucleus. 2018;9(1):216–226. 29517398 10.1080/19491034.2018.1449498 PMC5973257

[105] Sylvius N, Bonne G, Straatman K, *et al*. MicroRNA expression profiling in patients with lamin A/C-associated muscular dystrophy. Faseb J. 2011;25(11):3966–3978. 21840938 10.1096/fj.11-182915

[106] Bhattacharjee P, Banerjee A, Banerjee A, et al. Structural alterations of Lamin A protein in dilated cardiomyopathy. Biochemistry. 2013;52(24):4229–4241. 23701190 10.1021/bi400337t

[107] Über Zelltheilung C,R. Morph Jb. Vol. 10. 1885. p. 214–330.

